# Dexamethasone acutely suppresses the anabolic SNAT2/SLC38A2 amino acid transporter protein in L6‐G8C5 rat skeletal muscle cells

**DOI:** 10.1096/fba.2020-00076

**Published:** 2020-10-21

**Authors:** Safia Blbas, Emma Watson, Heather Butler, Jeremy Brown, Terence P. Herbert, Cordula M. Stover, Alan Bevington, Nima Abbasian

**Affiliations:** ^1^ Department of Respiratory Sciences University of Leicester Leicester UK; ^2^ Department of Cardiovascular Sciences University of Leicester Leicester UK; ^3^ John Walls Renal Unit University Hospitals of Leicester Leicester UK; ^4^ School of Pharmacy University of Lincoln Lincoln UK

**Keywords:** dexamethasone, glucocorticoid, skeletal muscle, SLC38A2, SNAT2, ubiquitin‐proteasome pathway

## Abstract

Chronic metabolic acidosis plays a role in cachexia by enhancing total proteolysis in skeletal muscle. Glucocorticoid also triggers proteolysis and plays a permissive role in the effect of acidosis. The System A amino acid transporter SNAT2/SLC38A2 is ubiquitously expressed in mammalian cells including muscle, performing Na^+^‐dependent active import of neutral amino acids, and is strongly inhibited by low pH. Exposure of rat skeletal muscle cell line L6‐G8C5 to low pH rapidly inhibits SNAT2 transport activity and enhances total proteolysis rate. Pharmacological inhibition or silencing of SNAT2 also enhances proteolysis. This study tests the hypothesis that the glucocorticoid dexamethasone (DEX), like low pH, inhibits SNAT2 activity in L6‐G8C5 myotubes, thus contributing to total proteolysis. Incubation with 500 nM DEX for 4 h reduced the System A amino acid transport rate to half the rate in control cultures. This inhibition depended on glucocorticoid receptor‐mediated gene transcription, but SNAT2 mRNA levels were unaffected by DEX. In contrast, the SNAT2 protein assessed by immunoblotting was significantly depleted. The co‐inhibitory effects of DEX and low pH on System A transport activity were additive in stimulating total proteolysis. In keeping with this mechanism, DEX’s inhibitory effect on SNAT2 transport activity was significantly blunted by the proteasome inhibitor MG132. Proof of principle was achieved in similar experiments using recombinant expression of a GFP‐tagged SNAT2 fusion protein in HEK293A cells. It is concluded that DEX acutely depletes the SNAT2 transporter protein, at least partly through proteasome‐dependent degradation of this functionally important transporter.

## 1 INTRODUCTION

Wasting of skeletal muscle during chronic catabolic illness is a common and clinically serious condition accompanying a wide range of illnesses, such as cancer, multiorgan failure during sepsis, cardiac insufficiency, chronic obstructive pulmonary disease, and chronic kidney disease (CKD), and is widely regarded as an important factor that negatively affects the long‐term survival of such patients.^1^ Atrophy of skeletal muscle fibers occurs as a result of an impairment of protein metabolism: partly through impaired protein synthesis but more importantly through enhanced proteolysis, most notably through the ubiquitin‐proteasome pathway (UPP).^2^ While chronic inflammation may be an important and widely applicable stimulus for this enhanced proteolysis,^3^ specific metabolic defects such as metabolic acidosis may play a part, especially in advanced CKD in which metabolic acidosis accompanies renal failure.^4^

System A amino acid transporters^5^ are solute transporter proteins which perform Na^+^‐dependent active transport of neutral amino acids (notably L‐glutamine^6^) into mammalian cells. They are characterized by their strong pH dependence, showing significant inhibition within a few minutes of exposure to low extracellular pH (probably as a direct consequence of protonation of the transporter)^7^; and by their ability to transport the synthetic N‐methylated amino acid methylaminoisobutyrate (MeAIB).^8^ These transporters are members of the SLC38 gene family^9^ of solute carrier proteins, and one member of this family (SLC38A2—also known as sodium‐coupled neutral amino acid transporter 2, SNAT2, SAT2, or ATA2) is ubiquitously expressed in mammalian cells, including skeletal muscle.^10^

On exposure of the rat skeletal muscle cell line L6‐G8C5 to a low extracellular pH to model the effects of metabolic acidosis in vitro, these cells show inhibition of System A amino acid transport activity resulting in the depletion of intracellular free amino acids, impaired anabolic signaling through the mTORC1^11^ and PI3K/Akt^12^ signaling pathways and impaired total protein synthesis^11^ and an enhanced total proteolysis rate^12^ resulting in net wasting of the total protein pool in the cells. Selective silencing of SLC38A2/SNAT2 using small interfering RNAs induces similar changes in free amino acids, mTORC1 and PI3K/Akt signaling and total protein metabolism, confirming that SNAT2 is the dominant isoform of System A transporter expressed in L6‐G8C5 cells^11^, ^12^ and suggesting that SNAT2 is a probable mediator of the muscle protein wasting observed in skeletal muscle during metabolic acidosis in vivo.

Glucocorticoids exert a complex series of effects on skeletal muscle cells. In addition to their well‐documented anti‐inflammatory effects, glucocorticoids in excess act as a stimulus for muscle wasting, impaired total protein synthesis,^13^ and UPP activation,^14^ especially in Type II skeletal muscle fibers.^14^, ^15^, ^16^ Glucocorticoids can also promote cell survival^17^ and muscle growth,^18^ for example through the glucocorticoid‐activated protein kinase SGK1.^18^ In addition to the protein catabolic effects of glucocorticoid alone, glucocorticoid also plays an important permissive role in activating total proteolysis in rat skeletal muscle in response to metabolic acidosis in vivo.^19^ The normal increase in muscle total proteolysis observed in rats which had been rendered acidotic by administration of ammonium chloride was abolished by adrenalectomy (thus strongly diminishing glucocorticoid synthesis) and was restored by the synthetic glucocorticoid dexamethasone (DEX).^19^ Furthermore, both in experimental animals and in humans,^19^, ^20^, ^21^ metabolic acidosis is accompanied by an increase in the circulating concentration of glucocorticoid. This may partly explain why acidotic patients with CKD show an elevated level of glucocorticoid which may worsen their catabolic state.^22^, ^23^

While the pH sensitivity of System A transporters is well documented, the effect of glucocorticoid on such transporters is unclear. The preceding evidence that SNAT2 may mediate the protein catabolic effects of low pH on L6‐G8C5 cells, and that coupling exists in vivo between the effects of metabolic acidosis and glucocorticoid, raises the important question of whether SNAT2 also mediates some protein catabolic effects of glucocorticoid in skeletal muscle cells. The present study was therefore designed to test the hypothesis that:

1. Glucocorticoid downregulates the expression of the SNAT2 transporter protein in L6‐G8C5 myotubes, and
2. that this downregulation leads to functionally important inhibition of System A amino acid transport activity in the plasma membrane, culminating in free amino acid depletion and enhanced proteolysis.

The initial experimental objective was to test this hypothesis using the synthetic glucocorticoid DEX in the L6‐G8C5 rat skeletal muscle cell line which has previously been used extensively^11^, ^12^, ^24^, ^25^, ^26^ as a model for regulation of SNAT2 in skeletal muscle.

## 2 MATERIALS AND METHODS

### 2.1 Materials

DEX and all drugs and biochemicals were obtained from Sigma‐Aldrich (Gillingham, UK) unless otherwise stated. DEX, RU38486, and SGK1 inhibitor were dissolved in dimethylsulfoxide (DMSO) before addition to experimental medium. The final resulting concentration of DMSO in the medium (up to 7 × 10^−4^% vol/vol) was also added to control cultures. Vanadate was purchased in the form of sodium orthovanadate and was treated as described previously^27^ to remove other vanadium species before use. Dephostatin and protein tyrosine phosphatase 1B (PTP1B) inhibitor (Calbiochem 539741^28^) were obtained from Merck/Calbiochem (Nottingham, UK). Drugs were applied to cultures at pharmacologically active final concentrations and incubation times which had previously been validated for vanadate,^29^ dephostatin,^30^ PTP1B inhibitor,^28^ MG132,^17^ RU38486,^17^ and SGK1 inhibitor (GSK650394).^31^ RU38486 and actinomycin D were incubated with cells for 7 h. SGK1 inhibitor was preincubated with the cells for 1 h as described previously^31^: control cultures were also preincubated with DMSO vehicle.

## 3 METHODS

### 3.1 Cell culture and incubations

L6 rat skeletal myoblasts (subclone G8C5) were obtained from the European Collection of Animal Cell Cultures (ref. 9212111) and were used at passage number 5‐20. Cells were propagated in Dulbecco's modified Eagle medium (DMEM—Invitrogen, Paisley, UK ref. 11880) with 5 mM D‐glucose and pyruvate, supplemented with 10 mg/l phenol red (Sigma), 100 U/ml penicillin G, 100 µg/ml streptomycin, 2 mM L‐glutamine, and 10% vol/vol heat‐inactivated fetal bovine serum (FBS). After 72 h the confluent cells were fused to form myotubes by incubating in Fusion Medium comprising Minimum Essential Medium (MEM) (Invitrogen ref. 21090) supplemented with 100 U/ml penicillin G, 100 µg/ml streptomycin, 2 mM L‐glutamine, and 2% vol/vol FBS. Fresh Fusion Medium was added after 2 days. After a further 2 days the myotubes were used for experimental incubations. Stock cultures were periodically screened to confirm the absence of mycoplasma using a LookOut^®^ mycoplasma PCR detection kit (Sigma‐Aldrich) according to the manufacturer's instructions.

Human Embryonic Kidney cells subclone 293A (HEK293A) were obtained from Thermo Fisher Scientific, Loughborough, UK (ref. R70507) and were used at passage number 3‐21. Cells were propagated in growth medium comprising high‐glucose DMEM (Sigma‐Aldrich ref. D6429) supplemented with 100 U/ml penicillin G, 100 µg/ml streptomycin and 10% vol/vol FBS on collagen I coated plates.

Unless otherwise stated, all experimental incubations (including those with HEK293A cells) were performed in modified Fusion Medium in which FBS had been replaced with 2% vol/vol heat‐inactivated dialyzed fetal bovine serum (DFBS, Invitrogen ref. 26400). An additional 8 mM NaHCO_3_ was added to achieve a pH of 7.4 under a 5% CO_2_ atmosphere. For experiments requiring medium at pH 7.1, the 8 mM NaHCO_3_ supplement was replaced with equimolar NaCl to maintain a constant Na^+^ concentration, and 6.3 mmol HCl was added per liter of medium. When experimental incubations were complete, cell proteins were extracted by homogenizing cells in lysis buffer to generate whole cell lysates.^32^ Lysis buffer comprised 10 mM beta glycerophosphate, 1 mM EDTA, 1 mM EGTA, 50 mM Tris‐HCl pH 7.5, 1 mM sodium orthovanadate, 50 mM sodium fluoride, 1 mM benzamidine, 0.2 mM phenylmethylsulfonyl fluoride, 1 µg/ml pepstatin A, 1 µg/ml leupeptin hemisulfate, 0.1% vol/vol beta mercaptoethanol, and 1% vol/vol Triton X‐100 detergent. In some experiments cells were homogenized in buffer without detergent followed by ultracentrifugation to generate a 170,000 g membrane fraction.^33^

### 3.2 Plasmids and transfection

SNAT2 cDNA was cloned by PCR amplification from a human SLC38A2 ORF Shuttle Clone (Source Bioscience, Nottingham, UK ref. OCAAo5051E1145D) into a pLEICS‐29 mammalian expression vector (University of Leicester Protein Expression Laboratory, Leicester, UK) (https://www2.le.ac.uk/colleges/medbiopsych/facilities‐and‐services/cbs/protex/available‐vectore/details‐of‐vectors/view) using the following primers: 5′‐CTACCGGACTCAGATCTCGAGATGAAGAAGGCCGAAATGGGACG‐3′ and, 5′‐TACCGTCGACTGCATGAATTCATGGCCACCTCCAGGTG‐3′. The resulting SNAT2 construct was confirmed by sequencing. It was designed to express, under the control of an SV40 viral promoter, a SNAT2—enhanced green fluorescent protein (eGFP) fusion protein with the following tandem linker plus eGFP tag sequence at its C‐terminus: EFMQSTVPRARDPPVAT‐eGFP EFMQSTVPRARDPPVAT‐eGFP. The resulting 8257 bp plasmid construct and the eGFP‐tagged SNAT2 fusion protein expressed from it are designated SNAT2‐eGFP. The corresponding 6696 bp ligated empty pLEICS‐29 vector (lacking the SNAT2 sequence) was used as a negative control. Plasmid transfection (using 1.9 µg of vector‐SNAT2 DNA construct, or 1.6 µg of equimolar empty vector DNA) was performed on 35 mm HEK293A cultures using a ProFection^®^ calcium phosphate transfection kit (Promega, Southampton, UK) according to the manufacturer's instructions. After 24 hours the transfection medium was discarded and replaced with HEK293A growth medium. Experiments were performed after a further 24 h. Expression of the construct or the empty vector was confirmed by monitoring eGFP fluorescence using an inverted Olympus IX81 motorized microscope with a Scan^R screening platform and a Cell^R imaging station. Quantification of fluorescence intensity was performed by a blinded observer using ImageJ software.

### 3.3 RNA methods

Total RNA was extracted from L6 myotubes using Trizol^®^ reagent (Invitrogen ref. 15596). From 1 µg of total RNA, cDNA was synthesized using an AMV Reverse Transcription System (Promega) according to the manufacturer's instructions. Real‐time PCR was performed using Power SYBER^®^Green PCR Master Mix (Thermo Fisher Scientific, Loughborough, UK) on an Applied Biosystems 7500 Fast Real‐Time PCR System (Applied Biosystems/Thermo Fisher Scientific, Loughborough, UK) with the rat primer sequences (Table 1).

**TABLE 1:** Primers used for qPCR

Primer		Sequence	Length	Amplicon size (bp)	NCBI Reference
PTP1B	F	TCGTCAGTGCAGGATCAGTG	20	102	NM_012637.2
	R	CTCCAATGTGCGTTTGGGTG	20		
SNAT2	F	GCTCATTCTCCCATTGTCAC	20	105	NM_181090.2
	R	TTGCAAATCACCACAATCAG	20		
Cyclophilin	F	CACCGTGTTCTTCGACATC	19	88	NM_017101.1
	R	TGCTGTCTTTGGAACTTTGTC	21		

Relative amounts of mRNA were normalized to the corresponding cyclophilin signal for each sample and relative expression is presented as (2^−ΔΔCT^).^34^

### 3.4 Immunoblotting and immunoprecipitation

Cell lysates or cell membranes (20 µg protein per lane) were subjected to SDS‐PAGE, and proteins were blotted onto Hybond ECL nitrocellulose membranes (GE Healthcare, Amersham, UK), followed by probing with primary antibodies against SNAT2 (a rabbit polyclonal raised against the 65 N‐terminal residues of rat SNAT2^10^); annexin II (Autogen Bioclear/Santa Cruz, Mile Elm, UK); P^Ser473^Akt, total Akt, GFP, P^Ser235/236^rpS6, P^Thr172^AMPK, and total AMPK (New England BioLabs, Hitchin, UK), and β‐Actin as a loading control (Abcam, Cambridge, UK). Bound primary antibody was detected using horseradish peroxidase (HRP)‐conjugated polyclonal goat anti‐mouse or goat anti‐rabbit immunoglobulins (DakoCytomation, Ely, UK) as appropriate; and HRP‐labeled proteins were detected by chemiluminescence using SuperSignal West Pico Chemiluminescent Substrate (Thermo Fisher Scientific). Band intensities were quantified using a ChemiDoc™ Touch Imaging System with Image Lab software v 5.2.1 (Bio‐Rad, Watford, UK).

Lysate samples containing 200 µg of total cell protein from cells expressing SNAT2‐eGFP, or eGFP from the empty control vector, were treated with GFP‐Trap^®^_MA beads (ChromoTek, Munich, FRG) according to the manufacturer's instructions to immunoprecipitate SNAT2‐eGFP and eGFP. The resulting GFP‐trapped proteins were separated by SDS‐PAGE followed by staining with RAPIDstain^TM^ reagent (Sigma‐Aldrich/Calbiochem), in‐gel trypsin digestion and analysis by liquid chromatography‐mass spectrometry (MS). MS was performed on an LTQ‐Orbitrap‐Velos‐ETD mass spectrometer with data analysis using a Mascot v2.2.04 search of the UniprotKB‐Swissprot database.

### 3.5 Analysis of free amino acid concentration and kinetics

Intracellular concentrations of free amino acids were measured as described previously.^11^ Briefly 35‐mm cultures were rapidly chilled on ice, rinsed to remove extracellular free amino acids, and deproteinized by scraping in ice‐cold 0.3 M perchloric acid, followed by centrifugation to remove precipitated protein. Perchloric acid in the soluble supernatant was neutralized and free amino acids in the resulting neutralized extract were determined on an Agilent 1100 high‐performance liquid chromatograph with a Zorbax Eclipse AAA column with o‐phthalaldehyde/3‐mercaptopropionate/9‐fluorenylmethylchloroformate precolumn derivatization and ultraviolet postcolumn detection. Data are expressed as nmoles of free amino acid per mg of acid‐precipitated cell protein determined by the Lowry method.^35^

System A amino acid transporter activity was assessed from the rate of uptake of the ^14^C‐labeled System A substrate MeAIB into intact cells. After incubation of the cells on 22‐mm culture wells in experimental media, cells were rinsed twice with 1 ml of Hepes‐buffered saline (HBS) comprising 140 mM NaCl, 2.5 mM MgSO_4_, 5.0 mM KCl, 1.0 mM CaCl_2_, 10 mg/L phenol red, and 20 mM Hepes acid titrated to pH 7·4 at room temperature with 0.5 M NaOH: (for transport assays at pH 7.1, the NaOH addition was reduced accordingly). Then 500 µl of HBS was added to each well. The transport experiment was started by adding to this an aliquot of α‐[1‐^14^C]‐methylaminoisobutyrate (^14^C‐MeAIB; NEN‐Du Pont/Perkin Elmer, Seer Green, UK; 3.70 MBq/ml; specific radioactivity 50 pCi/pmol) to give a final concentration of 10 µM ^14^C‐MeAIB in the culture well. In some culture wells an excess (10 mM) of unlabeled MeAIB (Sigma‐Aldrich) was also present in the well along with the ^14^C‐MeAIB as a negative control to assess nonspecific binding of radioactivity. The cultures with ^14^C‐MeAIB were incubated at 20°C for exactly 5 min, and then immediately placed on ice to stop transport activity. Cultures were rinsed with 3 × 1 ml of ice‐cold 0.9% w/v NaCl to remove extracellular radioactivity, followed by scraping of the cell monolayer in 200 µl of 0.05 M NaOH and then digestion at 70°C for 30 min and counting of the resulting lysate on an LKB 1219 liquid scintillation counter with quench correction to determine disintegrations per minute (dpm). The ^14^C‐MeAIB dpm count in the nonspecific binding control cultures was subtracted from the count in the other cultures to determine net ^14^C‐MeAIB transport into the cells.

The total proteolysis rate was measured in intact cells by prelabeling cell proteins with ^3^H‐L‐phenylalanine (^3^H‐L‐Phe) (NEN‐Du Pont/Perkin Elmer) and measuring the rate of release of acid‐soluble radioactivity into the culture medium as described previously,^36^ expressing the result as log_10_ of the percentage of the total initial cellular ^3^H per hour.^36^

### 3.6 Statistical analysis

**FIGURE 1:**
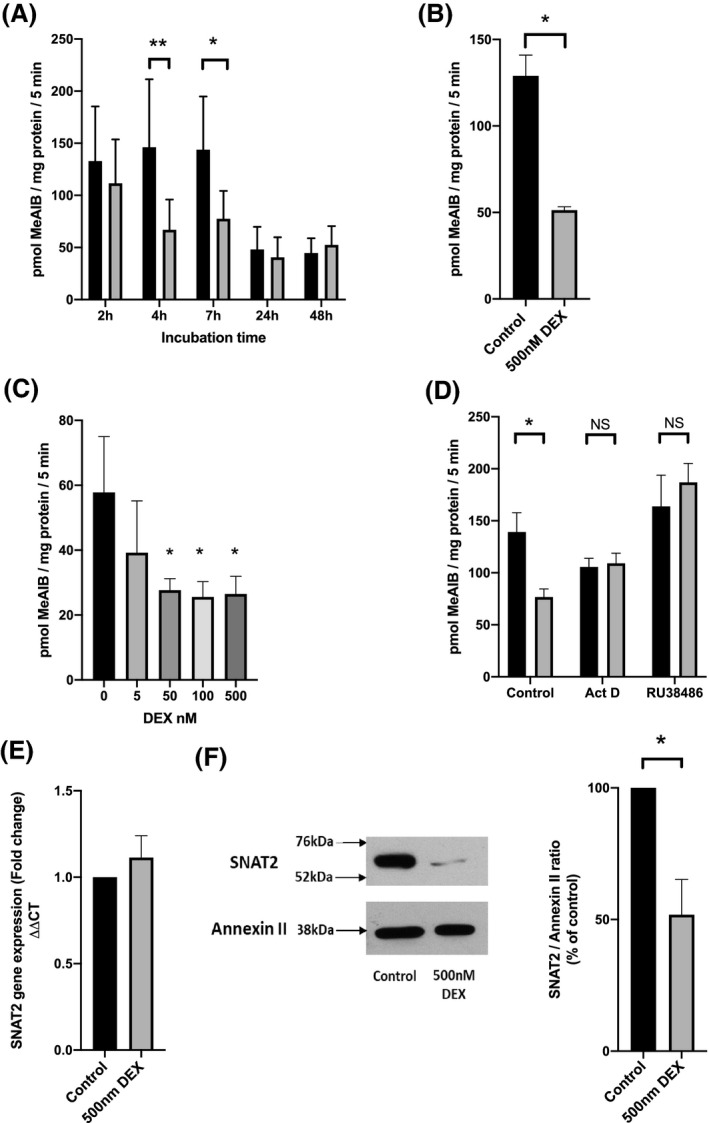
Effect of dexamethasone (DEX) on SNAT2 System A transporter activity and expression in L6‐G8C5 myotubes. A, Time course of the effect of DEX on System A transporter activity. Black bars control; gray bars 500 nM DEX. (*p < 0.05; **p < 0.01 vs. the corresponding control, n = 6); B, Effect of 4 h of incubation with 500 nM DEX on System A transporter activity in serum‐free medium after 15 h preincubation without serum (*p < 0.02, n = 3); C, Dose dependence of the effect of DEX on System A transporter activity (*p < 0.05 vs. control without DEX, n = 3); D, Effect of 7 h incubation with transcription inhibitor actinomycin D (Act D, 1 mM) or glucocorticoid receptor antagonist RU38486 (5 mM) on System A transporter activity. Black bars control; gray bars 500 nM DEX. (*p < 0.05 vs. corresponding cultures with DEX, n = 3); E, Determination by qRT‐PCR of SNAT2 mRNA after 4 h of incubation with 500 nM DEX, with cyclophilin as reference gene, n = 3; F, Corresponding effect of 500 nM DEX on SNAT2 protein expression determined by immunoblotting from a 170,000 g membrane fraction: (left) representative immunoblots showing Annexin II as reference protein. (right) densitometry. Black bar control; gray bar 500 nM DEX (*p < 0.05 vs. control, n = 5)

Data were analyzed using GraphPad Prism 8 and are presented as the mean ±SEM derived from at least n = 3 independent experiments. Normal distribution of data was tested using the Shapiro–Wilk test. Data showing non‐Gaussian distribution were log‐transformed before further analysis. Comparison of control data with a single test condition was performed by paired Student's *t* test. Paired densitometry data are presented as the ratio of the intensity for the protein of interest/housekeeping protein, expressed as a % of the corresponding ratio under control conditions, and were analyzed by ratio paired t‐test. Experiments involving multiple comparisons were analyzed by repeated measures ANOVA, followed by Tukey's multiple comparisons test. Multiple comparison densitometry experiments (which showed wide differences in variance between experimental groups) were analyzed by Friedman's nonparametric ANOVA. Experiments involving two experimental variables (e.g., DEX and time in Figure 1A) were analyzed by two‐way repeated measures ANOVA, followed by Dunnett's multiple comparisons test.

## 4 RESULTS

### 4.1 DEX inhibits system A amino acid transport in L6‐G8C5 myotubes

Incubation with 500 nM DEX for 4 h in medium with 2% serum reduced System A amino acid transport rate (which is mediated by SNAT2^11^) to 46.9 ± 2.0% of that in the control which had been incubated with DMSO vehicle (n = 6). This effect persisted at 7 hours but was no longer detectable after 24 h (Figure 1A). The control value was observed to decline after 24 h, possibly owing to transient stimulation of the transport rate by a serum component in the fresh medium that had been added at the start of the experiment. To test the possibility that 500 nM DEX was acting solely by blocking the stimulatory effect of a serum factor, the incubations at 4 h were repeated using serum‐free experimental medium after a 15‐h period of serum starvation (Figure 1B). This yielded an inhibitory effect of DEX identical to that previously seen (Figure 1A), indicating that this was a direct effect of DEX on the cells and not an indirect consequence of an effect of serum. To avoid apoptosis that may follow complete serum deprivation,^17^ all subsequent experiments were performed under 2% serum conditions.

Following 4 h of incubation with DEX, significant inhibition of transport was observed with as little as 50 nM DEX (Figure 1C). Furthermore, the effect of 500 nM DEX on transport was abolished by incubation with the glucocorticoid receptor antagonist RU38486 (Figure 1D) and by actinomycin D (Figure 1D) indicating that transport inhibition was mediated by a classical glucocorticoid receptor acting through a transcriptional mechanism. However, assay of SNAT2 mRNA by qRT‐PCR (Figure 1E) detected no direct transcriptional effect on the SNAT2 gene itself. In contrast, the SNAT2 protein assessed by immunoblotting was significantly depleted by incubation with DEX (Figure 1f).

### 4.2 Co‐inhibitory effects of DEX and low pH on transport activity are additive

**FIGURE 2:**
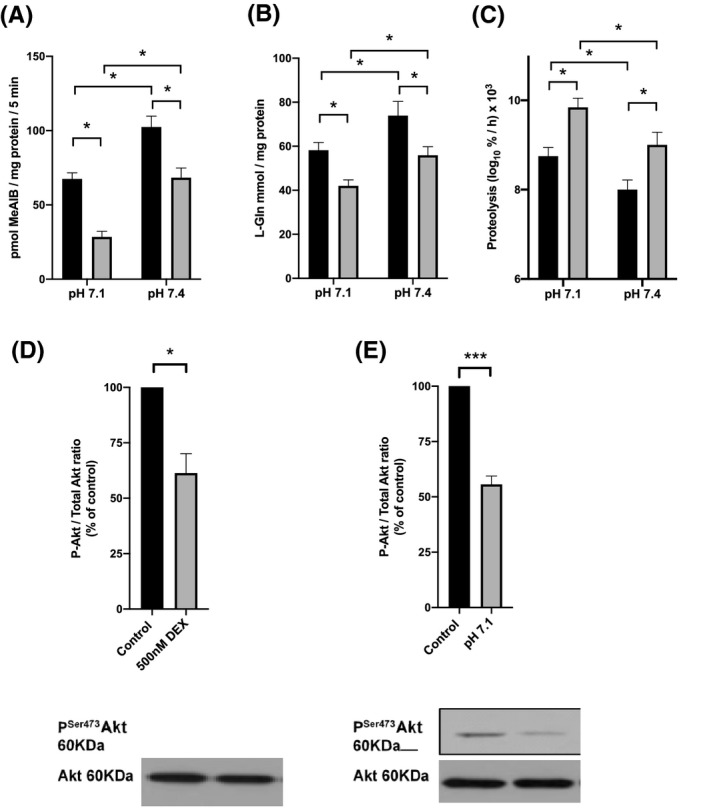
Combined effects of 500 nM dexamethasone (DEX) and a pH 7.1 extracellular acid load on L6‐G8C5 myotubes. A, System A transporter activity after 7 h with 500 nm DEX (gray bars) or control medium (black bars). At the end of the 7 h incubation, transporter activity was assayed immediately in Hepes‐buffered saline adjusted to a pH of 7.1 or 7.4 (see Methods). (*p < 0.05, n = 3); B, Intracellular L‐glutamine concentration after 7 h with 500 nm DEX (gray bars) or control medium (black bars). The culture medium comprised MEM at pH 7.1 or pH 7.4 with 2% vol/vol dialyzed fetal bovine serum. (*p < 0.05, n = 3); C, Global proteolysis rate measured in myotubes in which cell proteins had been prelabeled with ^3^H‐L‐phenylalanine. The data show the rate of release of ^3^H‐L‐phenylalanine into the medium from cultures as in (B) over the time interval t = 4‐7 h. (*p < 0.05, n = 4); d), E, Parallel comparison of the effects of incubation as in (B) with 500 nM DEX or low pH (7.1) on phospho‐activation of Akt assessed by immunoblotting. Bottom panels show representative blots. Top panels show corresponding densitometry (*p < 0.02, ***p < 0.001 n = 5)

The significantly reduced transport activity induced by 500 nM DEX after 4‐7 h (Figure 1A) is similar in magnitude to the acute inhibitory effect of lowering the extracellular pH from 7.4 to 7.1,^7^, ^37^ a low pH that has previously been used to model the effects of clinical metabolic acidosis.^11^, ^19^, ^38^ In view of the strong coupling reported in vivo between the catabolic effects of acidosis and glucocorticoid on skeletal muscle,^19^ and the concurrence of an elevated circulating concentration of glucocorticoid with acidosis in vivo,^19^, ^20^, ^21^ the combined effect of DEX and low pH was investigated. The inhibitory effects of DEX and low pH on transport activity were approximately additive (Figure 2A) and resulted in a corresponding additive depletion effect on the concentration of free L‐glutamine (a major SNAT2 substrate^6^) inside the cells (Figure 2B). As selective inhibition or silencing of SNAT2 in these cells has previously been shown to activate total proteolysis through inhibition of Akt signaling,^12^ the inhibitory effect of DEX and its combined effect with low pH would be predicted to lead to a commensurate increase in total proteolysis accompanied by impaired phospho‐activation of Akt. This was confirmed (Figure 2C‐E) suggesting that, like the inhibitory effect of low pH on SNAT2, the effect of DEX on this transporter contributes to the glucocorticoid‐induced activation of total proteolysis that is observed in muscle in vivo.^13^, ^14^, ^15^, ^16^

### 4.3 Involvement of glucocorticoid‐sensitive phosphatases or kinases

**FIGURE 3:**
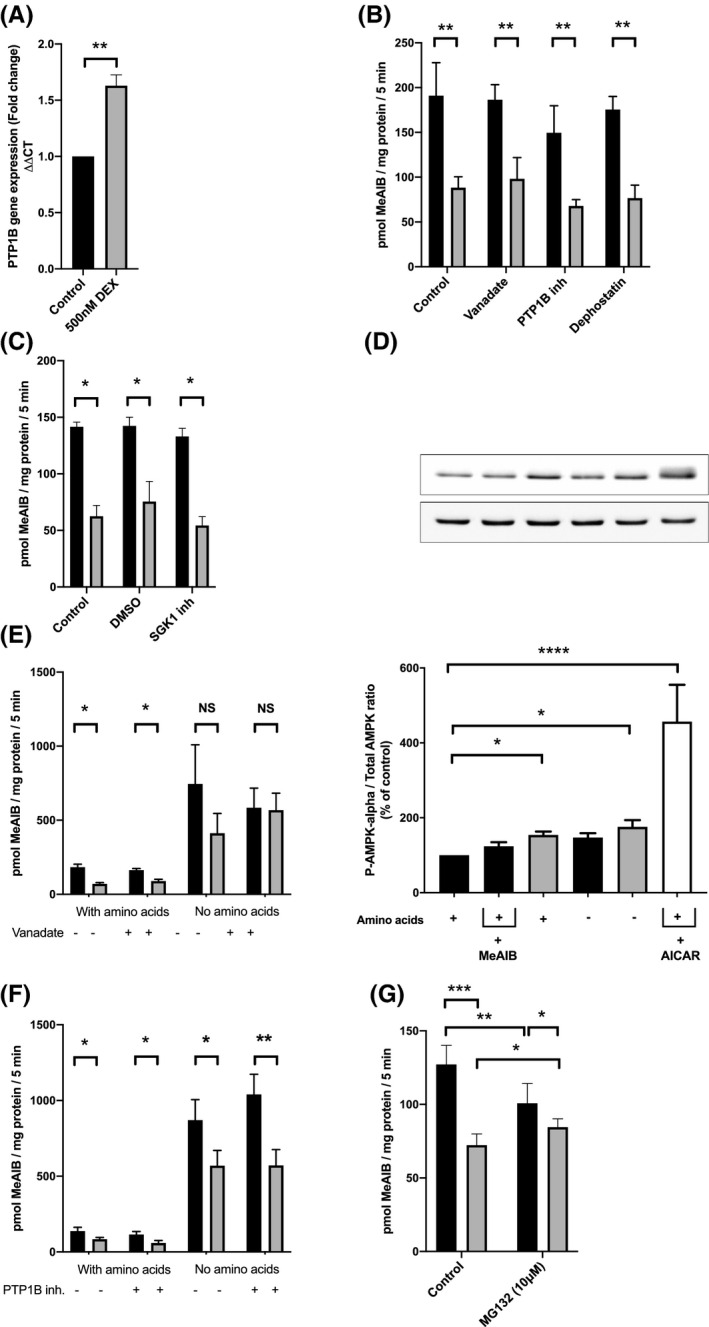
Pharmacological blockade of the effect of dexamethasone (DEX) on System A amino acid transport in L6‐G8C5 myotubes. Bars denote incubations without (black) or with (gray) DEX. A, Effect of 4 h incubations with 500 nM DEX on PTP1B mRNA assessed by qRT‐PCR with cyclophilin as reference gene, n = 3; (**p < 0.01, n = 4); B), C), E) and F) System A transporter activity after 4 h incubations with the specified combinations of 500 nM DEX, 100 μM vanadate, 20 mM PTP1B inhibitor, 20 mM Dephostatin, 103 nM SGK1 inhibitor or extracellular amino acid deprivation. (*p < 0.05; **p < 0.01, n = 3). In (C) cells were also preincubated for 1 h with 103 nM SGK1 inhibitor or vehicle (2 × 10^−4^% vol/vol DMSO). In (E) and (F) cells subjected to amino acid deprivation were also preincubated for 1 h in medium without amino acids. D, Phospho‐activation of AMPK by kinase LKB1 assessed by immunoblotting in response to the specified combinations of 500 nM DEX, 10 mM System A transport antagonist MeAIB, or extracellular amino acid deprivation. As a positive control, 750 μM AICAR was added to activate AMPK. Top panel shows representative immunoblots. Bottom panel shows corresponding densitometry. (*p < 0.05; ****p < 0.0001, n = 6). G, System A transporter activity after 4 h incubations with 10 mM proteasome inhibitor MG132. (*p < 0.05; **p < 0.01; ***p < 0.001, n = 4)

As DEX had no effect on the levels of SNAT2 mRNA (Figure 1E), to investigate how DEX impinges on SNAT2 activity the effect of DEX on glucocorticoid‐inducible phosphoprotein phosphatases and kinases was investigated. Indeed, it has previously been reported in skeletal muscle in vivo that glucocorticoid enhances gene expression of the phosphoprotein phosphatase PTP1B.^39^ This effect on PTP1B mRNA was confirmed here using DEX (Figure 3A) under the conditions that inhibited SNAT2 transport activity previously (Figure 1). However, neither the broad‐spectrum phosphoprotein tyrosine phosphatase inhibitors vanadate^29^ or dephostatin,^30^ nor a specific PTP1B inhibitor^28^ were able to block the inhibitory action of DEX on SNAT2 transport activity (Figure 3B). Similarly, even though the glucocorticoid‐inducible protein kinase SGK1 is known to be expressed in skeletal muscle,^18^ the specific SGK1 inhibitor GSK650394^31^ failed to blunt the action of DEX on SNAT2 (Figure 3C).

A further glucocorticoid‐modulated kinase is LKB1 which phosphorylates the key sensor of cellular energy status AMP‐dependent kinase (AMPK).^40^ LKB1 is downregulated by glucocorticoid,^40^ which consequently inhibits phospho‐activation of AMPK. However, using this phosphorylation of AMPK as a sensitive indicator of LKB1 activation, the expected DEX‐induced inhibition of AMPK phosphorylation was not observed in L6‐G8C5 myotubes, indeed an increase was detected (Figure 3D), suggesting that DEX was unlikely to be acting on SNAT2 through this pathway. As DEX had already been shown to deplete intracellular L‐glutamine (Figure 2B), a major metabolic fuel in cultured cells, which is consumed by L6 myotubes,^41^ it is possible that the postulated DEX‐induced decrease in AMPK phosphorylation had been masked here by AMPK phospho‐activation secondary to depletion of amino acid metabolic fuels. Therefore, the effect of total extracellular amino acid starvation, or competitive blockade of SNAT2 with an excess of its non‐metabolizable substrate MeAIB (which both strongly deplete intracellular L‐glutamine^11^), was also tested (Figure 3D). These positive controls involving amino acid depletion exerted only a weak stimulatory effect on AMPK phosphorylation, and inhibition of AMPK phosphorylation by DEX was still not observed even in medium devoid of SNAT2 amino acid substrates, (Figure 3D), confirming that DEX was unlikely to be acting on SNAT2 through LKB1 inhibition.^40^

### 4.4 Influence of amino acid starvation on DEX inhibition of SNAT2

In a number of cell types the most potent activator of SNAT2 expression and transport activity is extracellular amino acid starvation.^25^ This lack of extracellular amino acids is sensed both through cessation of amino acid transport through SNAT2 into the cells, and through a putative direct sensing of extracellular amino acid depletion by the SNAT2 protein.^25^, ^42^ To investigate whether DEX was inhibiting SNAT2 by blocking these amino acid sensing functions of the transporter, the effect of DEX on SNAT2 transport activity was tested in amino acid‐starved cells. As previously reported,^25^ amino acid starvation strongly activated transport activity in the absence of DEX (Figure 3E); and the previously observed inhibition of transport by DEX was slightly blunted by amino acid starvation (Figure 3E). Furthermore, even though vanadate alone only weakly blunted the action of DEX on SNAT2 activity (Figure 3B,E), when it was applied in combination with amino acid starvation, the inhibitory effect of DEX on SNAT2 was completely abolished (Figure 3E). In contrast, when amino acid starvation was applied in combination with a specific inhibitor of PTP1B (Figure 3F) or with dephostatin (data not shown), the inhibitory effect of DEX on transport was still observed, suggesting that vanadate's action in combination with amino acid starvation was unlikely to be occurring through inhibition of PTP1B or another phosphoprotein tyrosine phosphatase.

As the inhibition of SNAT2 transport activity by DEX was accompanied by depletion of SNAT2 protein (Figure 1F) but no depletion of SNAT2 mRNA (Figure 1E), this implied that depletion was occurring either through post‐transcriptional blockade of the translation of SNAT2 mRNA or through enhanced degradation of the SNAT2 protein. Amino acid starvation is known to inhibit SNAT2 protein degradation which occurs through the ubiquitin‐proteasome pathway (UPP),^26^ and vanadate is a potent inhibitor of ATP utilization by proteasomes,^43^, ^44^ implying that the observed synergism between amino acid starvation and vanadate in blocking the effect of DEX (Figure 3E) occurs through the UPP. UPP involvement was therefore tested directly by proteasome inhibition using the selective inhibitor MG132. As predicted, this significantly blunted the inhibitory effect of DEX on SNAT2 transport activity (Figure 3G).

### 4.5 Characterization of SNAT2‐EGFP expressed in HEK293A cells

To overcome the technical limitation that SNAT2 protein is expressed only at low level in cultured myotubes, necessitating sample concentration by isolating membranes prior to immunoblotting; and to assess the relevance of DEX’s effect on SNAT2 to human systems, an eGFP‐tagged human SNAT2 fusion protein was cloned and expressed in the readily transfected human cell line HEK293A. This also allowed quantification of the eGFP‐tagged SNAT2 fusion protein by fluorescence measurements, thus overcoming potential confounding factors associated with quantification by immunoblotting, for example variable antibody response to different SNAT2 glycoforms and/or variable proteolysis of the glycoforms during lysate preparation.

**FIGURE 4:**
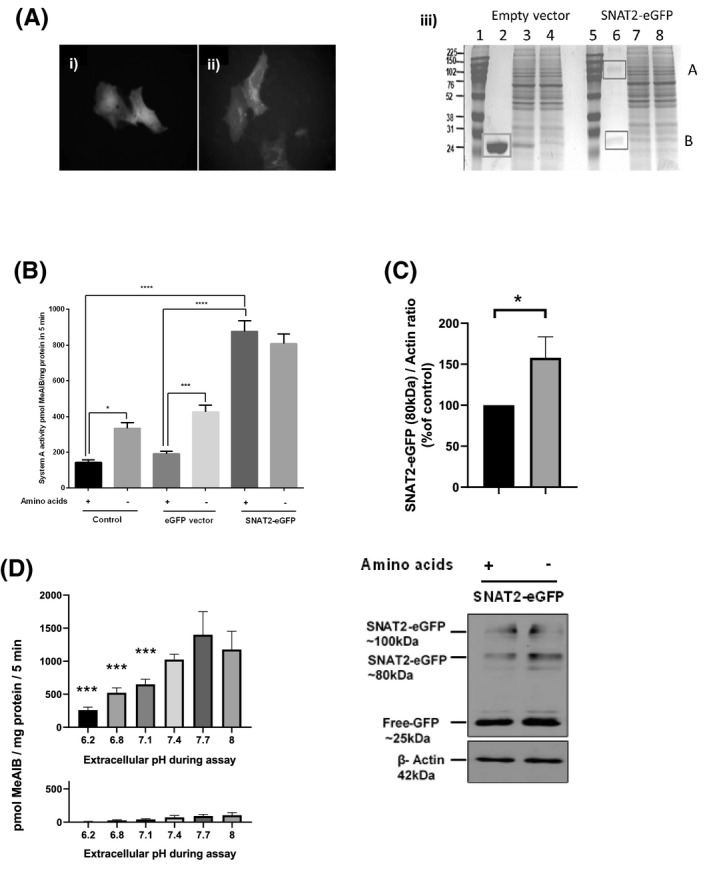
Characterization of SNAT2‐eGFP fusion protein expressed in HEK293A cells. A) Fluorescence microscopy showing eGFP expression 48 h after commencing transfection in i) cells transfected with ligated empty GFP pLEICS‐29 vector; ii) cells transfected with SNAT2‐eGFP construct. iii) SDS‐PAGE of GFP proteins immunoprecipitated from cultures as in (i) and (ii) by GFP‐trapping. Band A (lane 6) denotes SNAT2‐eGFP fusion protein. Band B (lanes 2 and 6) denotes free eGFP. Other lanes denote marker ladder (lanes 1 and 5), crude cell lysate (lanes 3 and 7), and first wash (lanes 4 and 8). B) System A transporter activity of cells transfected with SNAT2‐eGFP or empty control vector showing the effect of 4 h of extracellular amino acid starvation. (*p < 0.05, ***p < 0.001, ****p < 0.0001, n = 3). C, Detection of SNAT2‐eGFP fusion proteins in lysates from transfected HEK293A cells by immunoblotting with anti‐GFP antibody. Bottom panel—representative blots. Top panel densitometry performed on the light (80 kDa) SNAT2‐eGFP band. (*p < 0.05, n = 6). D, Effect of extracellular pH during the transport assay on System A transporter activity of cells transfected with SNAT2‐eGFP (top panel) or control HEK293A cultures (bottom panel). (***p < 0.001 vs. the pH 7.4 cultures, n = 3)

Fluorescence visualization of the tagged protein (Figure 4A) demonstrated a largely intracellular punctate, cytoplasmic and perinuclear distribution, consistent with the location expected for this membrane protein in the endoplasmic reticulum and Golgi region. In contrast, cells transfected with control empty vector showed a diffuse cytosolic distribution of eGFP fluorescence (Figure 4Ai vs. Aii). Isolation of the GFP‐tagged fusion protein by immunoprecipitation (GFP‐trapping) yielded a protein of approximately 100 kDa (Figure 4Aiii) which was confirmed by mass spectrometry to be SNAT2‐eGFP. To maximize the yield of the protein, SNAT2‐eGFP transfected cells were amino acid starved for 4 h to upregulate expression of the protein prior to eGFP‐trapping.

The characteristics of the expressed SNAT2‐eGFP protein were similar to those of SNAT2 in the L6‐G8C5 cell line. The fusion protein was shown to be a functionally active System A transporter leading to transport activity in transfected HEK293A cells which was fourfold higher than the endogenous transport activity observed in cells transfected with empty control vector (Figure 4B). Immunoblotting with anti‐GFP antibody (Figure 4C) detected a protein of the predicted molecular weight (~ 80 kDa) which was upregulated by amino acid starvation; accompanied by an apparent heavier isoform at ~100 kDa. These findings are consistent with an earlier report of approximately 75 and 90 kDa bands in anti‐GFP immunoblots of SNAT2‐eGFP transfected CHO‐K1 cells^45^ and of increased stabilization of the SNAT2 protein in amino acid‐starved cells.^25^

However, amino acid starvation in cells expressing the fusion protein yielded no further increase in transport activity in the plasma membrane beyond the high level already observed in cells replete with amino acids (Figure 4B). System A transport activity of the expressed fusion protein was acutely inhibited by low pH applied during the transport assay (Figure 4D top panel). Apparent pH dependence was also observed in the basal System A transport activity of nontransfected HEK293A cells. However, plotting the basal data on the same vertical scale as for the transfected cells (Figure 4D, bottom panel) showed that this basal pH sensitivity made a negligible contribution to the pH sensitivity of the transfected cells.

**FIGURE 5:**
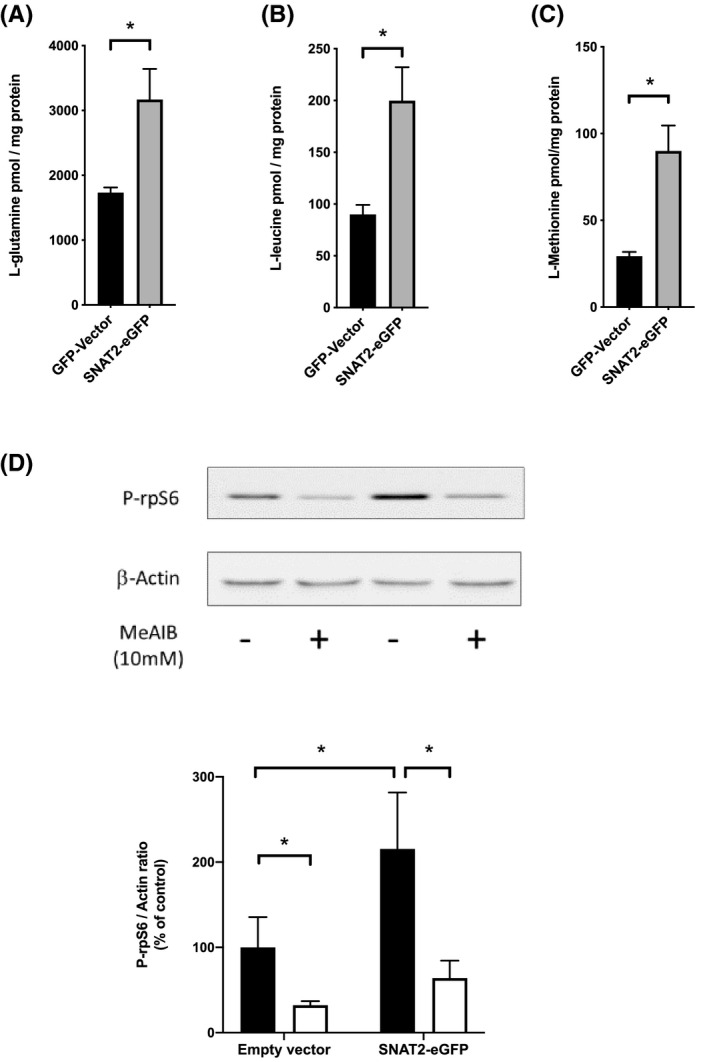
Effect of SNAT2‐eGFP fusion protein expression in HEK293A cells on free amino acid concentrations and amino acid signaling through mTORC1 to P‐rpS6. A), B), C) Free amino acid concentrations in HEK293A cells transfected with control vector or SNAT2‐eGFP. (*p < 0.05, n = 4); D) Amino acid signaling through SNAT2‐eGFP to P‐rpS6 via mTORC1. Top panel shows representative blots. Bottom panel shows corresponding densitometry. After transfection cultures were incubated for 2 h in control medium (black bars) or medium with 10 mM MeAIB (white bars) to block System A amino acid transport (*p < 0.05, n = 4)

Expression of the fusion protein led to a marked intracellular accumulation of free amino acids, notably L‐glutamine (Figure 5A), L‐leucine (Figure 5B), and L‐methionine (Figure 5C) whose intracellular concentrations have been shown previously to be regulated by SNAT2 expression in L6‐G8C5 cells.^11^ Consistent with earlier work,^11^ the resulting amino acid sensing through mTORC1 also enhanced System A‐dependent (i.e., MeAIB‐inhibitable) phosphorylation of rpS6 downstream from mTORC1 (Figure 5D).

### 4.6 Effect of DEX on human System A transporters expressed in HEK293A cells

**FIGURE 6:**
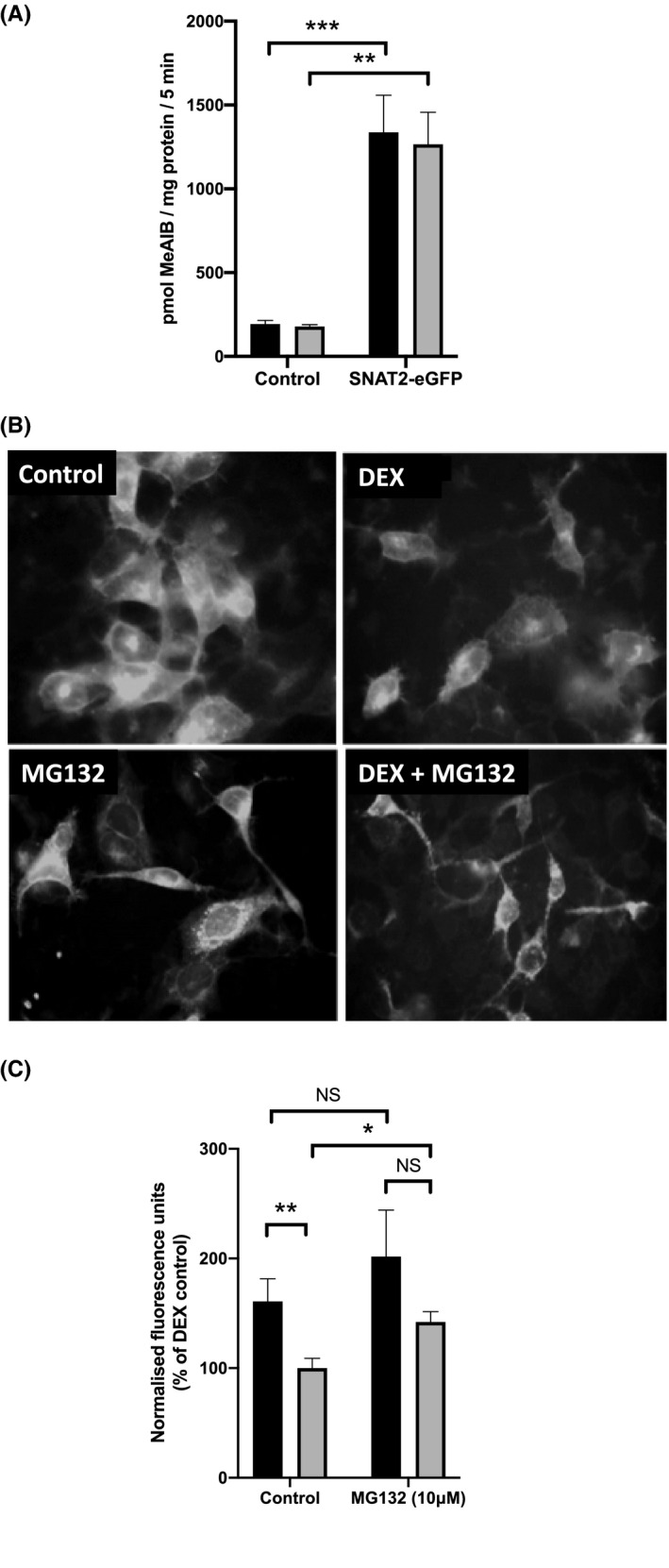
Effect of dexamethasone (DEX) on human System A amino acid transporters expressed in HEK293A cells. A, System A transporter activity of HEK293A cells transfected with SNAT2‐eGFP showing the effect of a 4 h incubation without (black) or with (gray) 500 nM DEX. (**p < 0.01; ****p < 0.0001, n = 3); B), C) The effect of 4 h of incubation with 500 nM DEX with or without 10 μM proteasome inhibitor MG132 on HEK293A cells transfected with SNAT2‐eGFP: B, representative fluorescence images; C, fluorescence intensity data quantified using ImageJ. Bars denote incubation without (black) or with (gray) 500 nM DEX. (*p < 0.05, **p < 0.01, NS – not significant, n = 8)

Unlike L6‐G8C5 myotubes (Figure 1), treatment of HEK293A cells (Figure 6A) with DEX for 4 h failed to inhibit the endogenous System A transport activity, nor did DEX inhibit the enhanced transport activity in HEK293A cells expressing SNAT2‐eGFP (Figure 6A), implying that the plasma membrane pool of human System A amino acid transporters might differ from that in rat (Figure 1) in its response to glucocorticoid. However, when the largely intracellular SNAT2‐eGFP fluorescence was quantified in cells expressing this fusion protein, a marked decrease in fluorescence was detected in response to DEX (Figure 6B,C), similar in magnitude to the downregulation of SNAT2 transport and protein expression that had been observed in L6‐G8C5 myotubes (Figure 1), and consistent with the hypothesis that intracellular SNAT2‐eGFP is degraded in response to DEX. As in L6‐G8C5 cells (Figure 3G) this was again confirmed by demonstrating reversal of the inhibitory effect of DEX by proteasome inhibition with MG132 (Figure 6B,C).

## 5 DISCUSSION

### 5.1 Transcription dependence of DEX‐induced SNAT2 suppression

The gene(s) through which DEX exerts its glucocorticoid receptor‐dependent transcriptional effect (Figure 1D) on SNAT2 transport activity are unknown, but these are unlikely to include the SNAT2/SLC38A2 gene itself. Even though the promoter region of the SNAT2 gene has recently been shown to contain a functionally important steroid response element regulated by estrogen,^46^ there is no evidence for a negative glucocorticoid response element in this region, and no decrease in SNAT2 mRNA was detected in response to DEX in the present study (Figure 1E) under conditions that gave a clear decrease in SNAT2 transport activity (Figure 1A) and protein expression (Figure 1F). It should also be emphasized that a negative glucocorticoid responsive element played no part in the depletion by DEX of the SNAT2‐eGFP fusion protein expressed in HEK293A cells (Figure 6B,C): the SNAT2 construct expressed in this system was controlled instead by an SV40 promoter.

It was also shown that DEX is unlikely to be acting on SNAT2 through transcription of the glucocorticoid‐dependent kinases SGK1 (Figure 3C) or LKB1 (Figure 3D) or the glucocorticoid‐dependent phosphoprotein tyrosine phosphatase PTP1B (Figure 3B). In view of the MG132 sensitivity of the DEX effect in Figure 3G and Figure 6B,C, and the abundant evidence that DEX can increase transcription of genes encoding proteins of the UPP in L6 myotubes,^47^, ^48^, ^49^ this seems the most likely pathway for DEX’s action, possibly through transcription of the E2 ubiquitin‐conjugating enzyme and the E3 ubiquitin ligases MAFbx/Atrogin‐1 and MuRF‐1.^47^, ^48^, ^49^ (The effect of DEX on the E3 ligase Nedd4‐2 which directly regulates SNAT2 as described below is currently unknown.) It should be noted, however that, unlike the time course observed in the present study (Figure 1A), these transcriptional effects only reach maximum after 24‐48 h.^47^, ^48^, ^49^ DEX effects have also been reported on the expression of ubiquitin and proteasome subunits themselves.^14^ However high doses of glucocorticoid are needed for this^50^; and because there is no glucocorticoid response element on the promoters of those genes, the mechanism is uncertain.

### 5.2 Dexamethasone downregulates the SNAT2 protein through a proteasome‐dependent pathway

It has previously been shown that the SNAT2 protein occurs in cells in at least two pools: a newly synthesized intracellular pool, and a mature glycosylated form that performs amino acid transport in the plasma membrane.^26^ The intracellular pool is preferentially degraded through direct ubiquitination of lysine residues on SNAT2 (mediated by E3 ligases such as Nedd4‐2^51^, ^52^) followed by proteasomal degradation.^26^, ^51^ From this two‐pool model, an effect of DEX on SNAT2 degradation should be observed more readily in the newly synthesized intracellular SNAT2‐eGFP pool inside transfected HEK293A cells, but not necessarily in the functionally active (i.e., amino acid transporting) pool in the plasma membrane. This is consistent with the observation of MG132‐responsive DEX‐induced degradation of eGFP‐tagged SNAT2 in Figure 6B,C, which occurred without a corresponding DEX‐induced decrease in the transport rate in the plasma membrane (Figure 6A). In contrast in L6‐G8C5 myotubes, the DEX‐induced total depletion of the SNAT2 protein detected by immunoblotting (Figure 1G) was accompanied by depletion of the transport‐mediating pool in the plasma membrane (Figure 1A). The mechanism that controls whether DEX and UPP‐mediated degradation of the intracellular pool leads to secondary depletion of the plasma membrane (transporter) pool as in L6‐G8C5 myotubes (Figure 1A), but not in HEK293A cells (Figure 6A), remains to be determined.

### 5.3 Influence of the SNAT2 C‐terminus on the response to DEX in HEK293A cells

A possible explanation for the lack of response of the plasma membrane transporter pool to DEX in SNAT2‐eGFP‐transfected HEK293A cells is that the presence of a C‐terminal tag domain in the protein disturbs the normal regulation of the transporter. Nevertheless, this transporter was clearly translocated efficiently into the plasma membrane where it was expressed as a functionally active transporter protein which actively accumulated free amino acids in the cells (Figure 5A‐C) and signaled to mTORC1 (Figure 5D) as previously described.^11^ The presence of a C‐terminal tag would be expected particularly to impair the normal sensing of low extracellular pH that is thought to occur through the C‐terminal His residue of SNAT2.^7^ However, when the pH dependence of the overexpressed SNAT2‐eGFP transport activity was assayed, the usual strong pH dependence was clearly observed (Figure 4D) indicating that the eGFP tag was not interfering with this function of the protein.

### 5.4 Biological significance of the effects of DEX on SNAT2

If the effect of DEX on SNAT2 reported here is functionally important, it should lead to a significant corresponding effect on total proteolysis and on the Akt signaling that controls it. It has also been known for many years that acidosis and elevation of glucocorticoid concentration occur together in vivo^19^, ^20^, ^21^ and that the enhancement of total proteolysis in skeletal muscle by metabolic acidosis in rats in vivo has an absolute requirement for glucocorticoid.^19^ To date, no molecular basis for the latter observation has been proposed. In view of previous evidence that SNAT2 activity and expression is an important determinant of total proteolysis rate in L6‐G8C5 myotubes,^12^ the observation in the present study that DEX and low pH reinforce each other's effects on SNAT2 transport activity (Figure 2A) with commensurate effects on anabolic Akt signaling (Figure 2D) and total proteolysis rate (Figure 2C) may partly explain the previously reported^19^ glucocorticoid dependence of skeletal muscle wasting in metabolic acidosis.

A related hypothesis for future research is that the DEX‐induced loss of SNAT2 protein reported here may be relevant to age‐related muscle wasting (sarcopenia). The loss of SNAT2 protein in aging mouse fast‐twitch muscle fibers cannot be explained by declining SNAT2 transcription,^53^ and possibly arises from increased degradation of the transporter, stimulated by the increased glucocorticoid secretion that occurs in aging^54^ and in sarcopenia.^55^

## 6 CONCLUSION

The data presented here demonstrate a clear and functionally important acute downregulation by glucocorticoid of the SNAT2 transporter protein. It is important to emphasize that this does not inevitably lead to inhibition of the active plasma membrane pool of transporter proteins responsible for System A amino acid transport activity. Even though a marked decrease in System A activity was observed in L6‐G8C5 cells (Figure 1A), this was not seen either in wild‐type or in SNAT2‐eGFP transfected HEK293A cells (Figure 6A). These observations, and reports elsewhere of the divergent effects of glucocorticoid on System A activity in nonmuscle cells, notably the stimulation reported in response to DEX in placenta^56^, ^57^ indicate that, in addition to driving SNAT2 proteolysis, glucocorticoid may also exert additional (possibly stimulatory) effects on the active System A transporter pool in the plasma membrane. The mechanism of this additional effect is unknown. It has recently been shown in placenta that translocation of intracellular SNAT2 to the plasma membrane on the actin cytoskeleton is regulated by a distinct pathway involving mTORC2 and Rho‐GTPases.^58^ However, the action of glucocorticoid on that pathway remains to be determined.

## CONFLICT OF INTEREST

The authors declare that they have no conflict of interest with the contents of this article.

## AUTHOR CONTRIBUTIONS

S.B., E.W., T.P.H., C.M.S., A.B., and N.A. designed the study; S.B., E.W., H.B., J.B., T.P.H., A.B., and N.A. performed the experiments; S.B., E.W., H.B., A.B., and N.A. involved in data analysis; S.B., E.W., A.B., and N.A. drafted the text and figures; S.B., E.W., H.B., J.B., T.P.H., C.M.S., A.B., and N.A. revised the paper. All the authors approved the final manuscript.

## Data Availability

The datasets used and/or analyzed during the current study are available from the corresponding authors on reasonable request.
